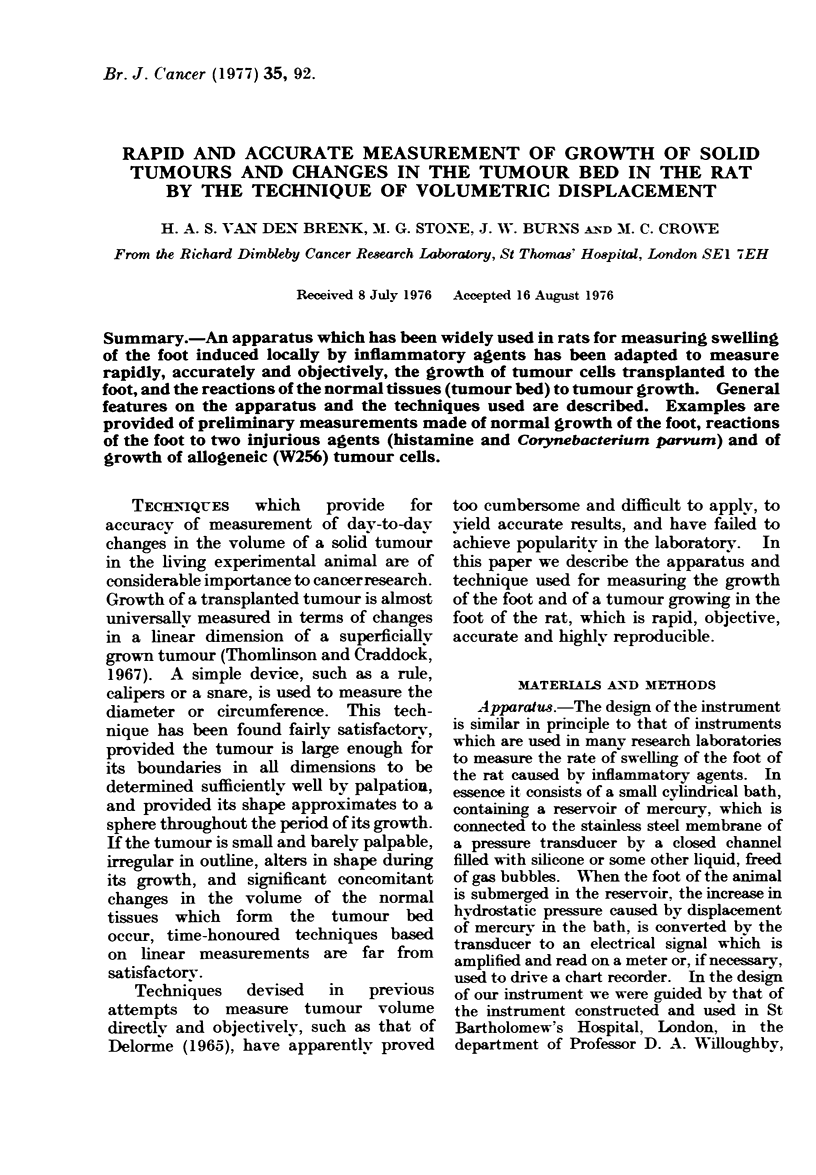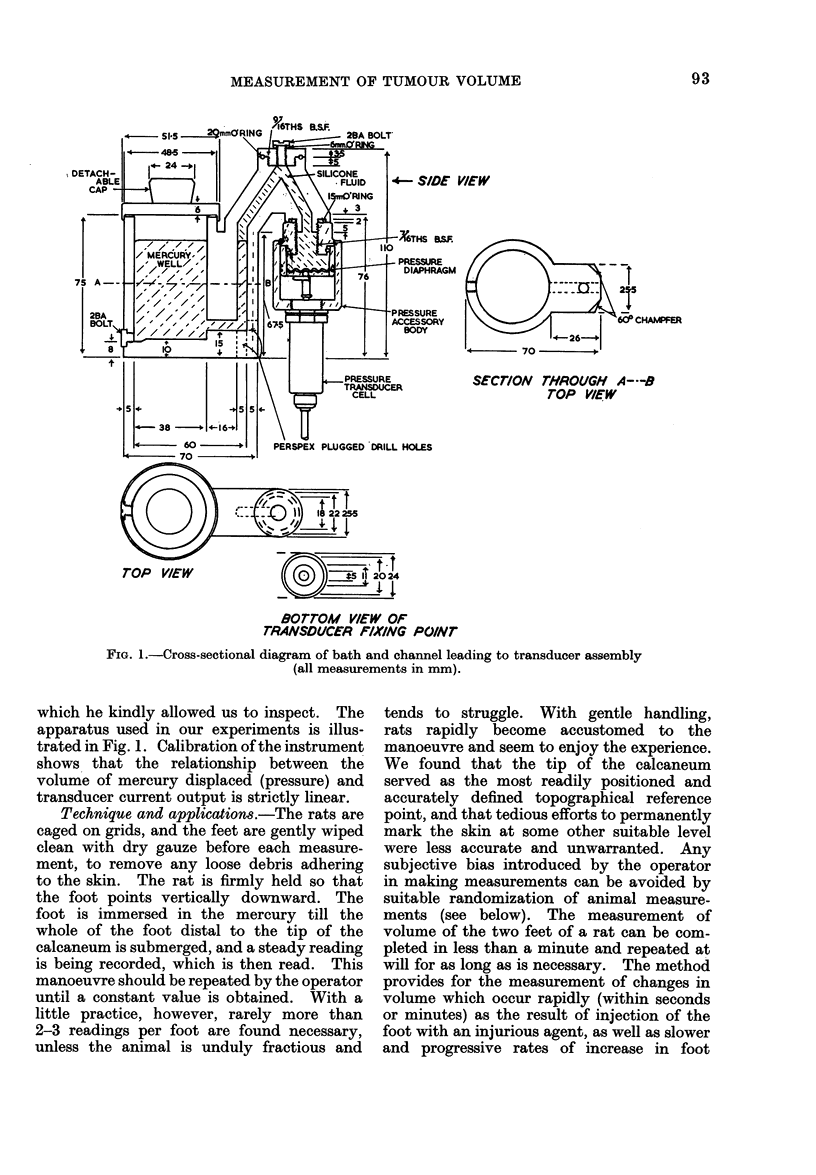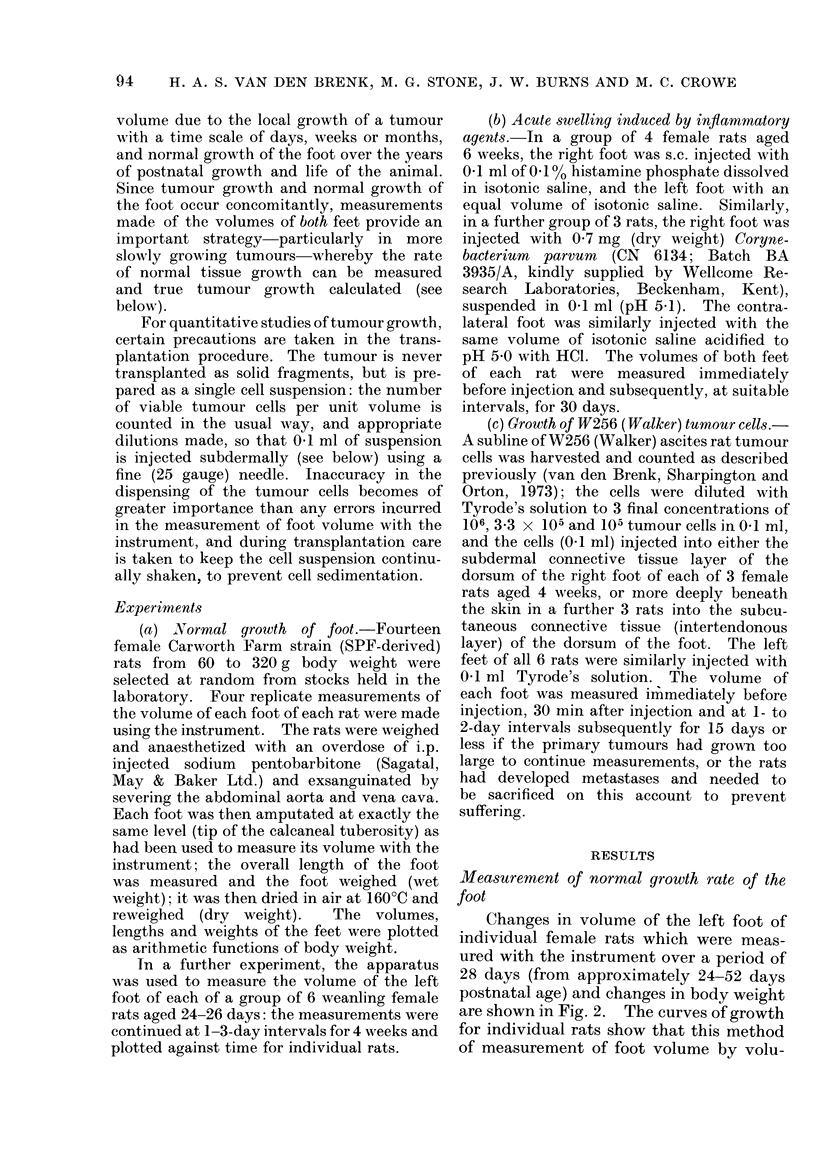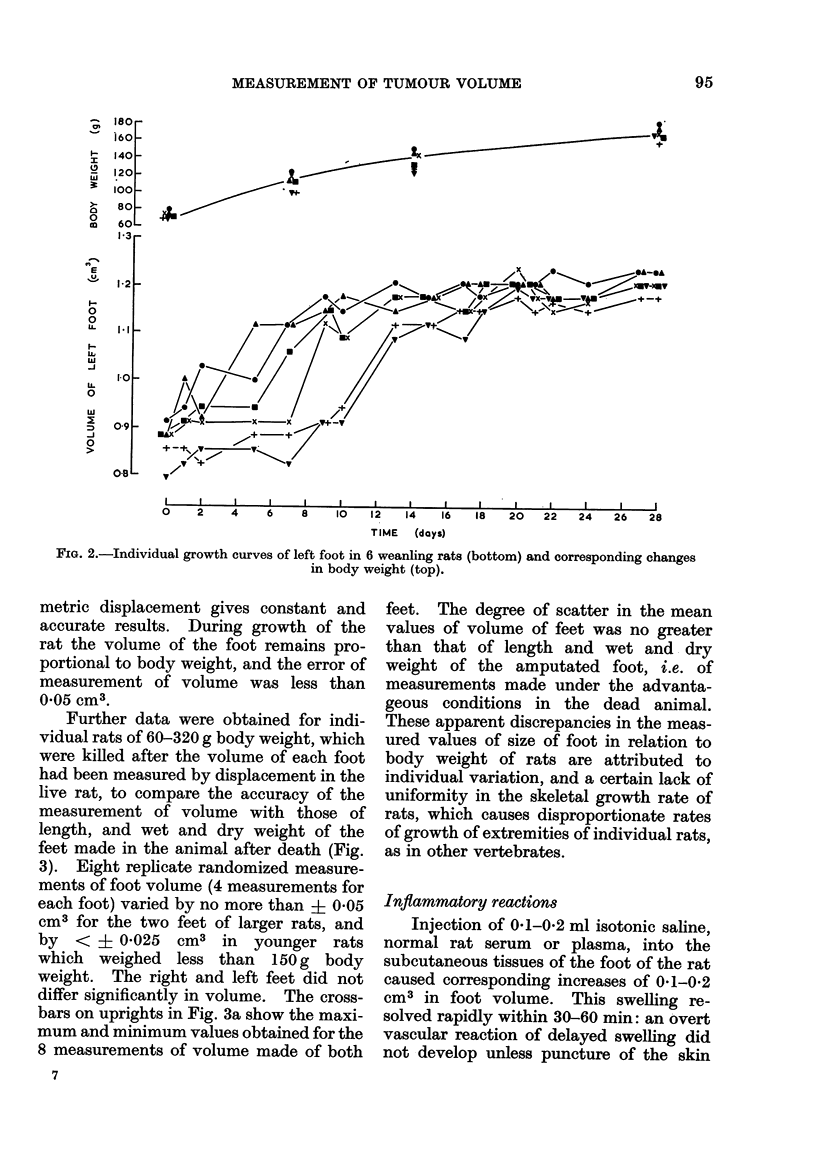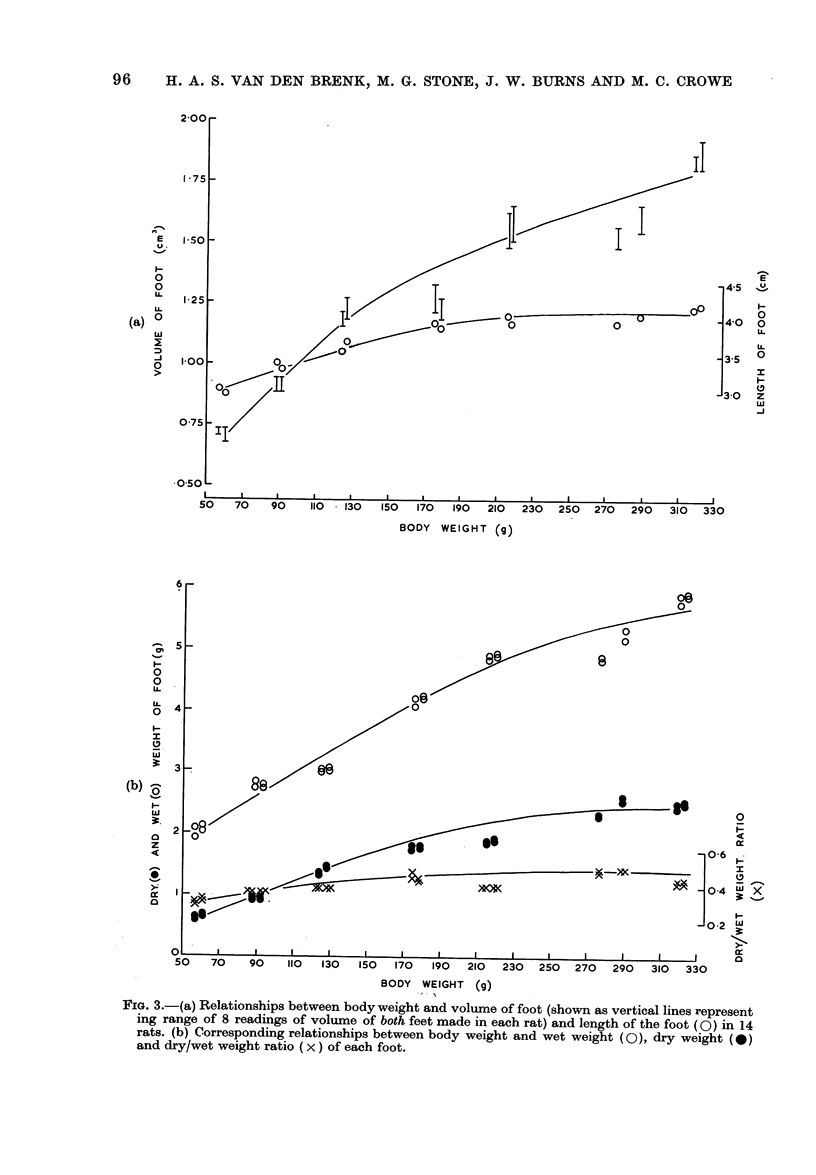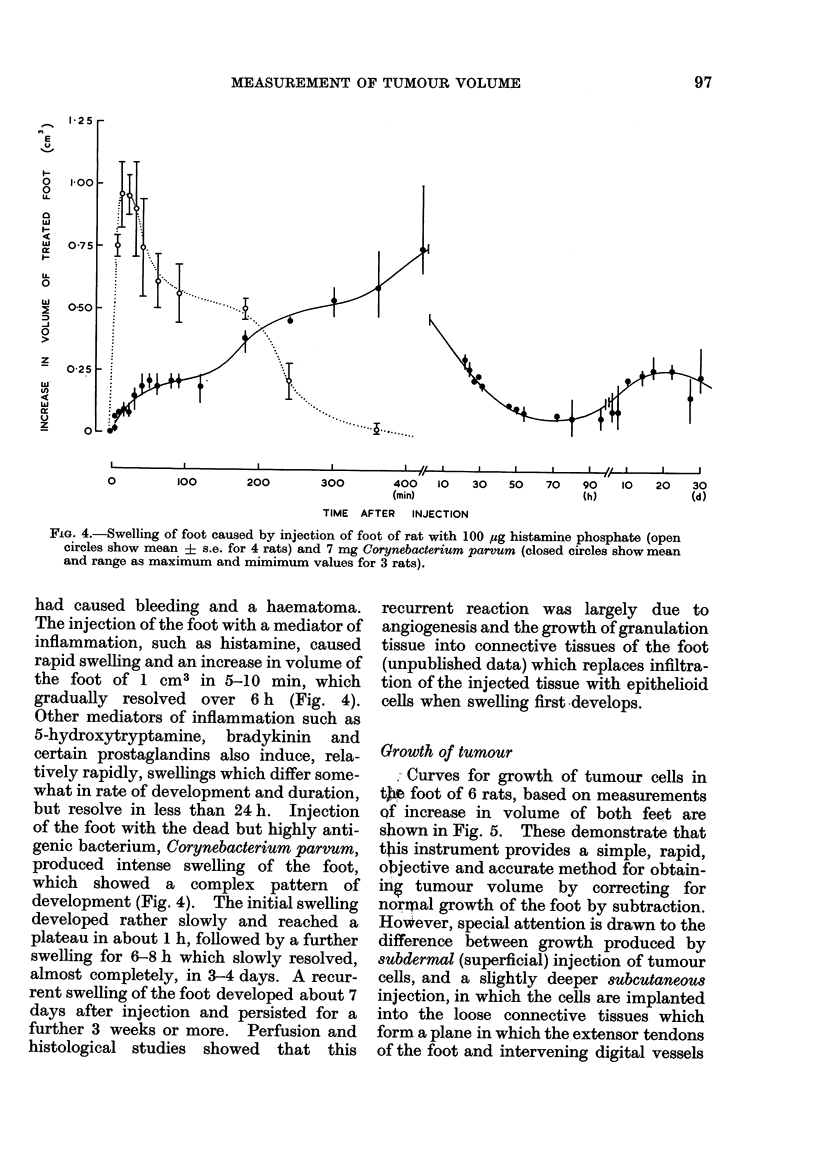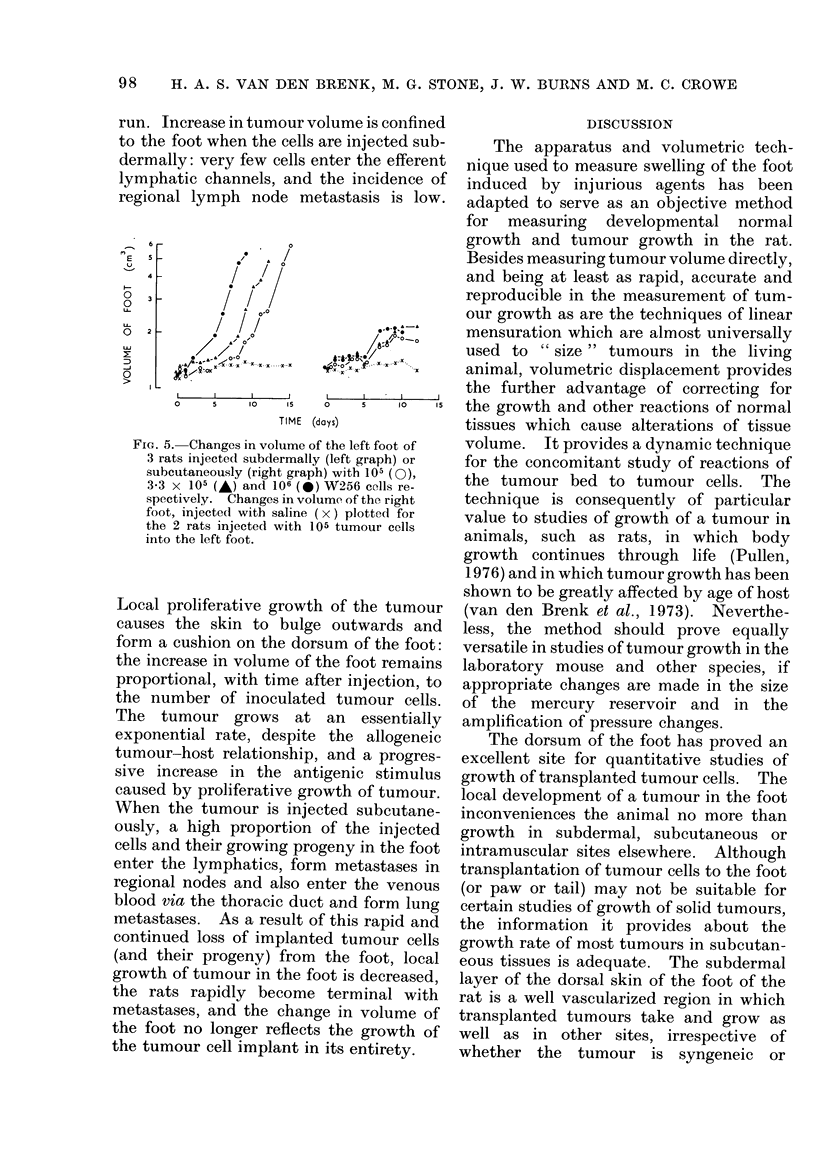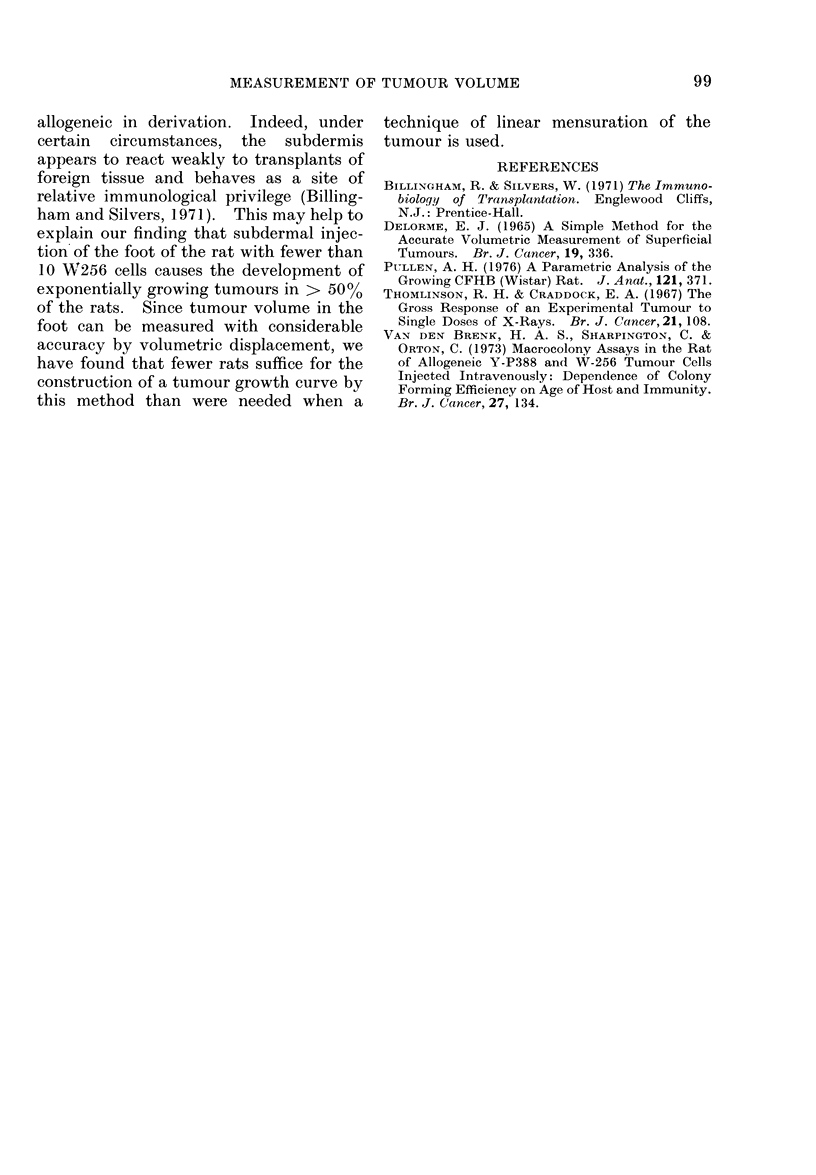# Rapid and accurate measurement of growth of solid tumours and changes in the tumour bed in the rat by the technique of volumetric displacement.

**DOI:** 10.1038/bjc.1977.8

**Published:** 1977-01

**Authors:** H. A. Van Den Brenk, M. G. Stone, J. W. Burns, M. C. Crowe

## Abstract

An apparatus which has been widely used in rats for measuring swelling of the foot induced locally by inflammatory agents has been adapted to measure rapidly, accurately and objectively, the growth of tumour cells transplanted to the foot, and the reactions of the normal tissues (tumour bed) to tumour growth. General features on the apparatus and the techniques used are described. Examples are provided of preliminary measurements made of normal growth of the foot, reactions of the foot to two injurious agents (histamine and Corynebacterium parvum) and of growth of allogeneic (W256) tumour cells.


*Br. J. Cancer* (1977) **35**, 92.

### RAPID AND ACCURATE MEASUREMENT OF GROWTH OF SOLID TUMOURS AND CHANGES IN THE TUMOUR BED IN THE RAT BY THE TECHNIQUE OF VOLUMETRIC DISPLACEMENT

H. A. S. VAN DEN BRENK, M. G. STONE, J. W. BURNS AND M. C. CROWE

*From the Richard Dimbleby Cancer Research Laboratory, St Thomas' Hospital, London SE1 7EH*

Received 8 July 1976 Accepted 16 August 1976

**Summary.—An apparatus which has been widely used in rats for measuring swelling of the foot induced locally by inflammatory agents has been adapted to measure rapidly, accurately and objectively, the growth of tumour cells transplanted to the foot, and the reactions of the normal tissues (tumour bed) to tumour growth. General features on the apparatus and the techniques used are described. Examples are provided of preliminary measurements made of normal growth of the foot, reactions of the foot to two injurious agents (histamine and *Corynebacterium parvum*) and of growth of allogeneic (W256) tumour cells.**

TECHNIQUES which provide for accuracy of measurement of day-to-day changes in the volume of a solid tumour in the living experimental animal are of considerable importance to cancer research. Growth of a transplanted tumour is almost universally measured in terms of changes in a linear dimension of a superficially grown tumour (Thomlinson and Craddock, 1967). A simple device, such as a rule, calipers or a snare, is used to measure the diameter or circumference. This technique has been found fairly satisfactory, provided the tumour is large enough for its boundaries in all dimensions to be determined sufficiently well by palpation, and provided its shape approximates to a sphere throughout the period of its growth. If the tumour is small and barely palpable, irregular in outline, alters in shape during its growth, and significant concomitant changes in the volume of the normal tissues which form the tumour bed occur, time-honoured techniques based on linear measurements are far from satisfactory.

Techniques devised in previous attempts to measure tumour volume directly and objectively, such as that of Delorme (1965), have apparently proved too cumbersome and difficult to apply, to yield accurate results, and have failed to achieve popularity in the laboratory. In this paper we describe the apparatus and technique used for measuring the growth of the foot and of a tumour growing in the foot of the rat, which is rapid, objective, accurate and highly reproducible.

### MATERIALS AND METHODS

*Apparatus.*—The design of the instrument is similar in principle to that of instruments which are used in many research laboratories to measure the rate of swelling of the foot of the rat caused by inflammatory agents. In essence it consists of a small cylindrical bath, containing a reservoir of mercury, which is connected to the stainless steel membrane of a pressure transducer by a closed channel filled with silicone or some other liquid, freed of gas bubbles. When the foot of the animal is submerged in the reservoir, the increase in hydrostatic pressure caused by displacement of mercury in the bath, is converted by the transducer to an electrical signal which is amplified and read on a meter or, if necessary, used to drive a chart recorder. In the design of our instrument we were guided by that of the instrument constructed and used in St Bartholomew's Hospital, London, in the department of Professor D. A. Willoughby,

FIG. 1.—Cross-sectional diagram of bath and channel leading to transducer assembly (all measurements in mm).

which he kindly allowed us to inspect. The apparatus used in our experiments is illustrated in Fig. 1. Calibration of the instrument shows that the relationship between the volume of mercury displaced (pressure) and transducer current output is strictly linear.

*Technique and applications.*—The rats are caged on grids, and the feet are gently wiped clean with dry gauze before each measurement, to remove any loose debris adhering to the skin. The rat is firmly held so that the foot points vertically downward. The foot is immersed in the mercury till the whole of the foot distal to the tip of the calcaneum is submerged, and a steady reading is being recorded, which is then read. This manoeuvre should be repeated by the operator until a constant value is obtained. With a little practice, however, rarely more than 2–3 readings per foot are found necessary, unless the animal is unduly fractious and tends to struggle. With gentle handling, rats rapidly become accustomed to the manoeuvre and seem to enjoy the experience. We found that the tip of the calcaneum served as the most readily positioned and accurately defined topographical reference point, and that tedious efforts to permanently mark the skin at some other suitable level were less accurate and unwarranted. Any subjective bias introduced by the operator in making measurements can be avoided by suitable randomization of animal measurements (see below). The measurement of volume of the two feet of a rat can be completed in less than a minute and repeated at will for as long as is necessary. The method provides for the measurement of changes in volume which occur rapidly (within seconds or minutes) as the result of injection of the foot with an injurious agent, as well as slower and progressive rates of increase in foot volume due to the local growth of a tumour with a time scale of days, weeks or months, and normal growth of the foot over the years of postnatal growth and life of the animal. Since tumour growth and normal growth of the foot occur concomitantly, measurements made of the volumes of *both* feet provide an important strategy—particularly in more slowly growing tumours—whereby the rate of normal tissue growth can be measured and true tumour growth calculated (see below).

For quantitative studies of tumour growth, certain precautions are taken in the transplantation procedure. The tumour is never transplanted as solid fragments, but is prepared as a single cell suspension: the number of viable tumour cells per unit volume is counted in the usual way, and appropriate dilutions made, so that 0·1 ml of suspension is injected subdermally (see below) using a fine (25 gauge) needle. Inaccuracy in the dispensing of the tumour cells becomes of greater importance than any errors incurred in the measurement of foot volume with the instrument, and during transplantation care is taken to keep the cell suspension continually shaken, to prevent cell sedimentation.

*Experiments*

(*a*) *Normal growth of foot.*—Fourteen female Carworth Farm strain (SPF-derived) rats from 60 to 320 g body weight were selected at random from stocks held in the laboratory. Four replicate measurements of the volume of each foot of each rat were made using the instrument. The rats were weighed and anaesthetized with an overdose of i.p. injected sodium pentobarbitone (Sagatal, May & Baker Ltd.) and exsanguinated by severing the abdominal aorta and vena cava. Each foot was then amputated at exactly the same level (tip of the calcaneal tuberosity) as had been used to measure its volume with the instrument; the overall length of the foot was measured and the foot weighed (wet weight); it was then dried in air at 160°C and reweighed (dry weight). The volumes, lengths and weights of the feet were plotted as arithmetic functions of body weight.

In a further experiment, the apparatus was used to measure the volume of the left foot of each of a group of 6 weanling female rats aged 24–26 days: the measurements were continued at 1–3-day intervals for 4 weeks and plotted against time for individual rats.

(*b*) *Acute swelling induced by inflammatory agents.*—In a group of 4 female rats aged 6 weeks, the right foot was s.c. injected with 0·1 ml of 0·1% histamine phosphate dissolved in isotonic saline, and the left foot with an equal volume of isotonic saline. Similarly, in a further group of 3 rats, the right foot was injected with 0·7 mg (dry weight) *Corynebacterium parvum* (CN 6134; Batch BA 3935/A, kindly supplied by Wellcome Research Laboratories, Beckenham, Kent), suspended in 0·1 ml (pH 5·1). The contralateral foot was similarly injected with the same volume of isotonic saline acidified to pH 5·0 with HCl. The volumes of both feet of each rat were measured immediately before injection and subsequently, at suitable intervals, for 30 days.

(*c*) *Growth of W256 (Walker) tumour cells.*—A subline of W256 (Walker) ascites rat tumour cells was harvested and counted as described previously (van den Brenk, Sharpington and Orton, 1973); the cells were diluted with Tyrode's solution to 3 final concentrations of $10^6$, $3·3 \times 10^5$ and $10^5$ tumour cells in 0·1 ml, and the cells (0·1 ml) injected into either the subdermal connective tissue layer of the dorsum of the right foot of each of 3 female rats aged 4 weeks, or more deeply beneath the skin in a further 3 rats into the subcutaneous connective tissue (intertendonous layer) of the dorsum of the foot. The left feet of all 6 rats were similarly injected with 0·1 ml Tyrode's solution. The volume of each foot was measured immediately before injection, 30 min after injection and at 1- to 2-day intervals subsequently for 15 days or less if the primary tumours had grown too large to continue measurements, or the rats had developed metastases and needed to be sacrificed on this account to prevent suffering.

### RESULTS

### *Measurement of normal growth rate of the foot*

Changes in volume of the left foot of individual female rats which were measured with the instrument over a period of 28 days (from approximately 24–52 days postnatal age) and changes in body weight are shown in Fig. 2. The curves of growth for individual rats show that this method of measurement of foot volume by volu-

FIG. 2.—Individual growth curves of left foot in 6 weanling rats (bottom) and corresponding changes in body weight (top).

metric displacement gives constant and accurate results. During growth of the rat the volume of the foot remains proportional to body weight, and the error of measurement of volume was less than 0·05 cm$^3$.

Further data were obtained for individual rats of 60–320 g body weight, which were killed after the volume of each foot had been measured by displacement in the live rat, to compare the accuracy of the measurement of volume with those of length, and wet and dry weight of the feet made in the animal after death (Fig. 3). Eight replicate randomized measurements of foot volume (4 measurements for each foot) varied by no more than $\pm$ 0·05 cm$^3$ for the two feet of larger rats, and by $< \pm$ 0·025 cm$^3$ in younger rats which weighed less than 150 g body weight. The right and left feet did not differ significantly in volume. The cross-bars on uprights in Fig. 3a show the maximum and minimum values obtained for the 8 measurements of volume made of both feet. The degree of scatter in the mean values of volume of feet was no greater than that of length and wet and dry weight of the amputated foot, *i.e.* of measurements made under the advantageous conditions in the dead animal. These apparent discrepancies in the measured values of size of foot in relation to body weight of rats are attributed to individual variation, and a certain lack of uniformity in the skeletal growth rate of rats, which causes disproportionate rates of growth of extremities of individual rats, as in other vertebrates.

*Inflammatory reactions*

Injection of 0·1–0·2 ml isotonic saline, normal rat serum or plasma, into the subcutaneous tissues of the foot of the rat caused corresponding increases of 0·1–0·2 cm$^3$ in foot volume. This swelling resolved rapidly within 30–60 min: an overt vascular reaction of delayed swelling did not develop unless puncture of the skin

FIG. 3.—(a) Relationships between body weight and volume of foot (shown as vertical lines representing range of 8 readings of volume of *both* feet made in each rat) and length of the foot (○) in 14 rats. (b) Corresponding relationships between body weight and wet weight (○), dry weight (●) and dry/wet weight ratio (×) of each foot.

FIG. 4.—Swelling of foot caused by injection of foot of rat with 100 μg histamine phosphate (open circles show mean ± s.e. for 4 rats) and 7 mg *Corynebacterium parvum* (closed circles show mean and range as maximum and mimimum values for 3 rats).

had caused bleeding and a haematoma. The injection of the foot with a mediator of inflammation, such as histamine, caused rapid swelling and an increase in volume of the foot of 1 $cm^3$ in 5–10 min, which gradually resolved over 6 h (Fig. 4). Other mediators of inflammation such as 5-hydroxytryptamine, bradykinin and certain prostaglandins also induce, relatively rapidly, swellings which differ somewhat in rate of development and duration, but resolve in less than 24 h. Injection of the foot with the dead but highly antigenic bacterium, *Corynebacterium parvum*, produced intense swelling of the foot, which showed a complex pattern of development (Fig. 4). The initial swelling developed rather slowly and reached a plateau in about 1 h, followed by a further swelling for 6–8 h which slowly resolved, almost completely, in 3–4 days. A recurrent swelling of the foot developed about 7 days after injection and persisted for a further 3 weeks or more. Perfusion and histological studies showed that this recurrent reaction was largely due to angiogenesis and the growth of granulation tissue into connective tissues of the foot (unpublished data) which replaces infiltration of the injected tissue with epithelioid cells when swelling first develops.

### *Growth of tumour*

Curves for growth of tumour cells in the foot of 6 rats, based on measurements of increase in volume of both feet are shown in Fig. 5. These demonstrate that this instrument provides a simple, rapid, objective and accurate method for obtaining tumour volume by correcting for normal growth of the foot by subtraction. However, special attention is drawn to the difference between growth produced by *subdermal* (superficial) injection of tumour cells, and a slightly deeper *subcutaneous* injection, in which the cells are implanted into the loose connective tissues which form a plane in which the extensor tendons of the foot and intervening digital vessels run. Increase in tumour volume is confined to the foot when the cells are injected subdermally: very few cells enter the efferent lymphatic channels, and the incidence of regional lymph node metastasis is low.

Fig. 5.—Changes in volume of the left foot of 3 rats injected subdermally (left graph) or subcutaneously (right graph) with $10^5$ (○), $3 \cdot 3 \times 10^5$ (▲) and $10^6$ (●) W256 cells respectively. Changes in volume of the right foot, injected with saline (×) plotted for the 2 rats injected with $10^5$ tumour cells into the left foot.

Local proliferative growth of the tumour causes the skin to bulge outwards and form a cushion on the dorsum of the foot: the increase in volume of the foot remains proportional, with time after injection, to the number of inoculated tumour cells. The tumour grows at an essentially exponential rate, despite the allogeneic tumour–host relationship, and a progressive increase in the antigenic stimulus caused by proliferative growth of tumour. When the tumour is injected subcutaneously, a high proportion of the injected cells and their growing progeny in the foot enter the lymphatics, form metastases in regional nodes and also enter the venous blood *via* the thoracic duct and form lung metastases. As a result of this rapid and continued loss of implanted tumour cells (and their progeny) from the foot, local growth of tumour in the foot is decreased, the rats rapidly become terminal with metastases, and the change in volume of the foot no longer reflects the growth of the tumour cell implant in its entirety.

### DISCUSSION

The apparatus and volumetric technique used to measure swelling of the foot induced by injurious agents has been adapted to serve as an objective method for measuring developmental normal growth and tumour growth in the rat. Besides measuring tumour volume directly, and being at least as rapid, accurate and reproducible in the measurement of tumour growth as are the techniques of linear mensuration which are almost universally used to " size " tumours in the living animal, volumetric displacement provides the further advantage of correcting for the growth and other reactions of normal tissues which cause alterations of tissue volume. It provides a dynamic technique for the concomitant study of reactions of the tumour bed to tumour cells. The technique is consequently of particular value to studies of growth of a tumour in animals, such as rats, in which body growth continues through life (Pullen, 1976) and in which tumour growth has been shown to be greatly affected by age of host (van den Brenk *et al.*, 1973). Nevertheless, the method should prove equally versatile in studies of tumour growth in the laboratory mouse and other species, if appropriate changes are made in the size of the mercury reservoir and in the amplification of pressure changes.

The dorsum of the foot has proved an excellent site for quantitative studies of growth of transplanted tumour cells. The local development of a tumour in the foot inconveniences the animal no more than growth in subdermal, subcutaneous or intramuscular sites elsewhere. Although transplantation of tumour cells to the foot (or paw or tail) may not be suitable for certain studies of growth of solid tumours, the information it provides about the growth rate of most tumours in subcutaneous tissues is adequate. The subdermal layer of the dorsal skin of the foot of the rat is a well vascularized region in which transplanted tumours take and grow as well as in other sites, irrespective of whether the tumour is syngeneic or allogeneic in derivation. Indeed, under certain circumstances, the subdermis appears to react weakly to transplants of foreign tissue and behaves as a site of relative immunological privilege (Billingham and Silvers, 1971). This may help to explain our finding that subdermal injection of the foot of the rat with fewer than 10 W256 cells causes the development of exponentially growing tumours in > 50% of the rats. Since tumour volume in the foot can be measured with considerable accuracy by volumetric displacement, we have found that fewer rats suffice for the construction of a tumour growth curve by this method than were needed when a technique of linear mensuration of the tumour is used.

### REFERENCES

Billingham, R. & Silvers, W. (1971) *The Immunobiology of Transplantation.* Englewood Cliffs, N.J.: Prentice-Hall.

Delorme, E. J. (1965) A Simple Method for the Accurate Volumetric Measurement of Superficial Tumours. *Br. J. Cancer*, **19**, 336.

Pullen, A. H. (1976) A Parametric Analysis of the Growing CFHB (Wistar) Rat. *J. Anat.*, **121**, 371.

Thomlinson, R. H. & Craddock, E. A. (1967) The Gross Response of an Experimental Tumour to Single Doses of X-Rays. *Br. J. Cancer*, **21**, 108.

Van den Brenk, H. A. S., Sharpington, C. & Orton, C. (1973) Macrocolony Assays in the Rat of Allogeneic Y-P388 and W-256 Tumour Cells Injected Intravenously: Dependence of Colony Forming Efficiency on Age of Host and Immunity. *Br. J. Cancer*, **27**, 134.